# Coding space-time stimulus dynamics in auditory brain maps

**DOI:** 10.3389/fphys.2014.00135

**Published:** 2014-04-08

**Authors:** Yunyan Wang, Yoram Gutfreund, José L. Peña

**Affiliations:** ^1^Dominick P. Purpura Department of Neuroscience, Albert Einstein College of MedicineBronx, NY, USA; ^2^The Rappaport Research Institute and Faculty of MedicineThe Technion, Haifa, Israel

**Keywords:** sound localization, adaptation, center-surround, acoustic motion, direction selectivity, maps

## Abstract

Sensory maps are often distorted representations of the environment, where ethologically-important ranges are magnified. The implication of a biased representation extends beyond increased acuity for having more neurons dedicated to a certain range. Because neurons are functionally interconnected, non-uniform representations influence the processing of high-order features that rely on comparison across areas of the map. Among these features are time-dependent changes of the auditory scene generated by moving objects. How sensory representation affects high order processing can be approached in the map of auditory space of the owl's midbrain, where locations in the front are over-represented. In this map, neurons are selective not only to location but also to location over time. The tuning to space over time leads to direction selectivity, which is also topographically organized. Across the population, neurons tuned to peripheral space are more selective to sounds moving into the front. The distribution of direction selectivity can be explained by spatial and temporal integration on the non-uniform map of space. Thus, the representation of space can induce biased computation of a second-order stimulus feature. This phenomenon is likely observed in other sensory maps and may be relevant for behavior.

Here we consider the emergence of tuning to temporally-dynamic stimulus features underlying motion selectivity in the owl's external nucleus of the inferior colliculus (ICx). Tuning in ICx displays a topography that corresponds with spatial coordinates (Knudsen and Konishi, 1978a). This map allows approaching ICx as a “retina” for auditory space. Thus, reverse-correlation methods such as the white-noise stimulation used by studies in vision (Chichilnisky, 2001; Recio-Spinoso et al., 2005) can be adapted to assess the selectivity of neurons to spatially-dynamic features of the auditory scene.

Motion detection requires integration over space and time. Below, we first examine integration over space, in the center-surround interactions of spatial receptive fields, and over time, in the history-dependent response properties of single cells. Subsequently, we address how the map topography influences tuning across the population. We conclude by discussing functional implications for coding acoustic motion direction.

## Surround suppression in auditory spatial receptive fields

**Figure 1:**
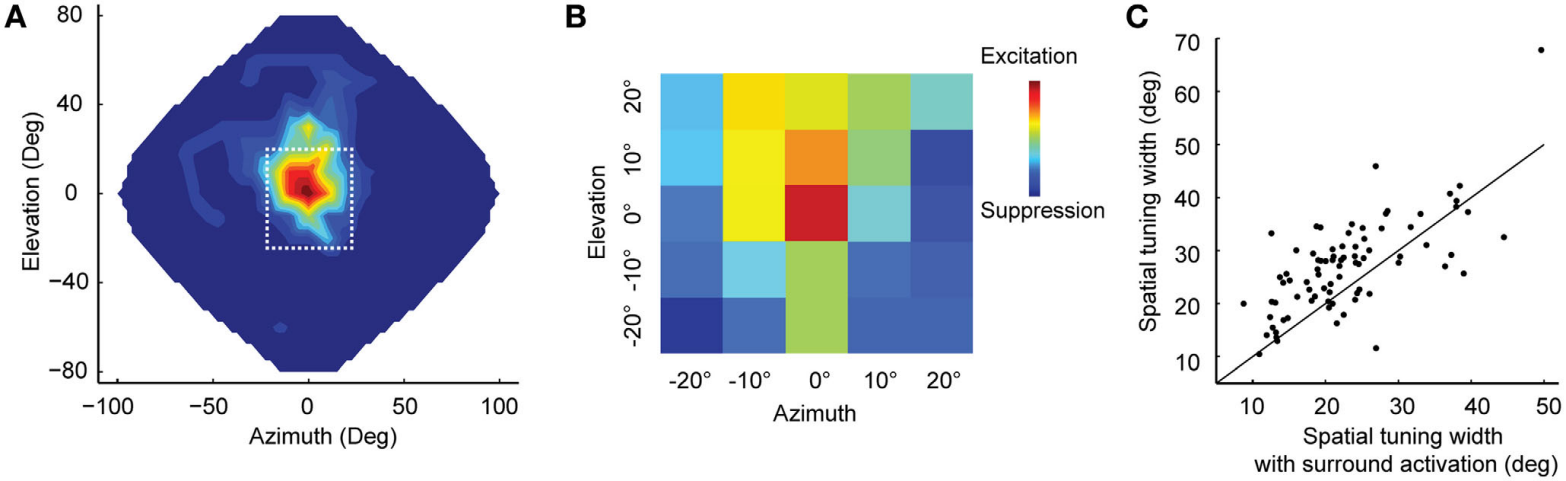
**Surround suppression in ICx. (A)** Example spatial receptive field of an ICx neuron. Firing rates were interpolated across 144 speakers covering frontal space. The dotted line indicates the area shown in **(B)**. **(B)** A subset of speakers was used to analyze the center-surround receptive field with white noise stimulation. Each colored box represents the cell's response to a speaker location. The receptive field showed excitation at the center flanked by suppression. **(C)** Surround activation sharpens the spatial tuning. For most cells (*n* = 81), the width of spatial-tuning curves was narrower when both center and surround were stimulated (points above the unity line) compared to when only the center was stimulated. Modified from Wang et al. (2012).

Experimental evidence is consistent with surround suppression in ICx (Knudsen and Konishi, 1978b; Fujita and Konishi, 1991). Topography within ICx allows the formation of lateral connections that correspond to neighboring relationships in auditory space. Recently, Wang et al. (2012) showed that simultaneous stimulation of the receptive-field center and surround of ICx neurons could result in up to 50% attenuation of response at the center compared to when the center was stimulated alone. This study used white-noise stimulation from concurrent random locations to measure the equivalent of the classical and extra-classical receptive fields in vision (Marmarelis and McCann, 1973; Chichilnisky, 2001; Recio-Spinoso et al., 2005) in space-specific neurons of ICx (Figures 1A,B). Surround suppression had a sharpening effect on spatial tuning at the center (Figure 1C), consistent with the narrowing of receptive fields observed using multiple concurrent sound sources (Bremen and Middlebrooks, 2013).

There is strong evidence that local GABAergic inhibition mediates surround suppression in sensory systems (Cook and McReynolds, 1998; Bloomfield and Xin, 2000; Völgyi et al., 2002; Sohn and Hallett, 2004; Foeller et al., 2005). Surround suppression is consistent with the sharpened spatial tuning in ICx during white noise stimulation (Wang et al., 2012), and with the broadening of tuning after the application of GABA antagonists (Fujita and Konishi, 1991; Mori, 1997; Zheng and Knudsen, 1999, 2001). It is also possible that GABA-mediated lateral inhibition in space originates upstream to ICx. In the avian auditory pathway, ICx is not the first stage in the pathway where localization cues are represented topographically. ITD, the primary cue for azimuth direction in barn owls, is mapped in the nucleus laminaris (Carr and Konishi, 1988, 1990; Carr and Boudreau, 1993; Carr et al., 2013), which projects topographically to the inferior colliculus (IC; Knudsen, 1983; Takahashi et al., 1984, 1989; Takahashi and Konishi, 1988; Carr and Boudreau, 1991). GABAergic transmission is conspicuously present at locations where spatial cues are encoded (Carr et al., 1989; Burger et al., 2005; Lu et al., 2005). Further, feedback connections may mediate surround suppression (Burger and Pollak, 2001). There are point-to-point projections from the optic tectum (OT) to the ICx involved in visual calibration of the auditory map (Luksch et al., 2000; Hyde and Knudsen, 2001, 2002). Lateral inhibition in OT could be carried back to ICx through these connections. Finally, recent evidence suggests that glycinergic inhibition may also play an important role in sound localization, and potentially could contribute to surround suppression (Kuo et al., 2009; Coleman et al., 2011; Fischl et al., 2013).

Mechanisms other than inhibitory projections may induce surround suppression in the auditory pathway. Because sound localization using ITD is based on cross-correlation (Blauert, 1997; Fischer et al., 2008), decreasing interaural correlation reduces the response (Albeck and Konishi, 1995; Saberi et al., 1998; Coffey et al., 2006). Interaural correlation is affected by the number of simultaneous sound sources from different locations and by the properties of the acoustic space (Blauert, 1997). A complex auditory scene can thus induce binaural decorrelation and decrease response when the surround is stimulated. However, decorrelation could not explain the asymmetry in surround suppression observed in Wang et al. (2012).

## History-dependent response: adaptation in ICx

**Figure 2:**
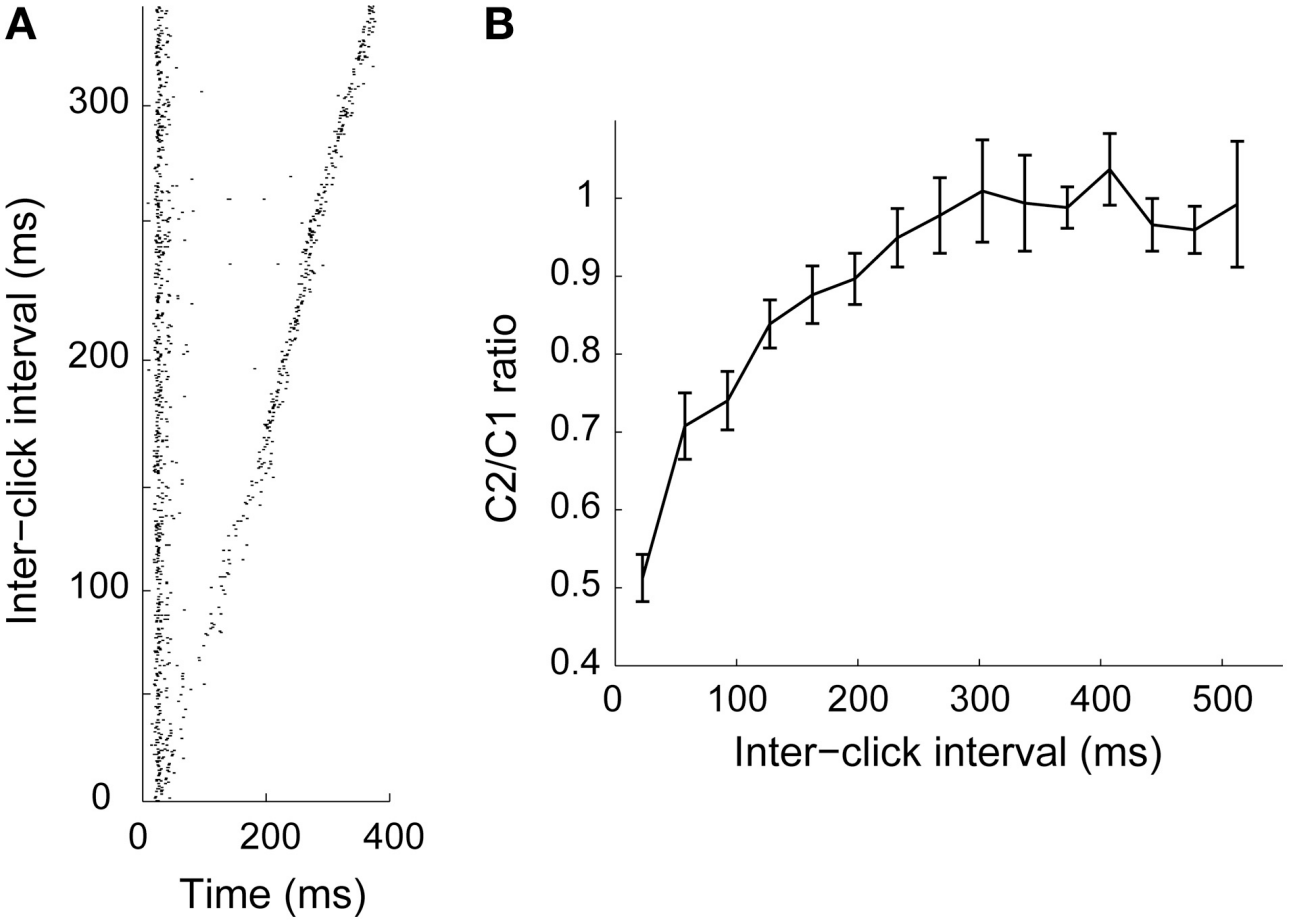
**Adaptation time course in ICx. (A)** Pairs of 1 ms clicks (C1 and C2) presented at various inter-click intervals. When the onset of the two clicks was close in time, response to the second click decreased relative to the first one. **(B)** Ratio between responses to C1 and C2 as a function of inter-stimulus interval (*n* = 44). Response to C2 was significantly attenuated when the interval was less than 300 ms. From Wang and Pena (2013).

Motion detection requires that the neural computation captures changes in location over time. Previously established motion detection models based on lateral excitation and inhibition meet this requirement (Hassenstein and Reichardt, 1958; Torre and Poggio, 1978). On the other hand, adaptation could also constitute a means for spatial integration over time, where the recovery time constitutes the duration of the “memory” in the system (Ulanovsky et al., 2004; Gutfreund and Knudsen, 2006). Similar to nuclei upstream to ICx, in the lateral shell and core of the inferior colliculus (Gutfreund and Knudsen, 2006; Singheiser et al., 2012), ICx neurons show adaptation that recovers in hundreds of milliseconds (Figure 2; Gutfreund and Knudsen, 2006; Wang and Pena, 2013). Recovery time from adaptation generally increases along the auditory pathway from tens of milliseconds in the auditory nerve (Harris and Dallos, 1979; Relkin and Turner, 1988) to hundreds of milliseconds in IC (Gutfreund and Knudsen, 2006; Netser et al., 2011; Singheiser et al., 2012), thalamus (Wehr and Zador, 2005) and the auditory cortex (Brosch and Schreiner, 1997; Ulanovsky et al., 2004; Wehr and Zador, 2005; Nelson et al., 2009). It has been suggested that the long time scale of adaptation plays a role in multisensory integration (Gutfreund and Knudsen, 2006), by permitting convergence of sensory modalities processed at different latencies (Bergan and Knudsen, 2009).

Several studies have alluded to synaptic depression as the underlying mechanism for the slow component in the recovery time from adaptation (Ulanovsky et al., 2004; Wehr and Zador, 2005; Gutfreund and Knudsen, 2006). Synaptic depression due to slowly re-activating T-type calcium channels has been demonstrated to play a role in forward suppression lasting several 100 ms (Bayazitov et al., 2013). Intracellular studies in the avian brainstem showed that short-term depression is prevalent in the auditory pathway (Kuba et al., 2002; Cook et al., 2003; MacLeod and Carr, 2007; MacLeod et al., 2007; MacLeod, 2011). In the context of spike-frequency adaptation, particular attention has been paid to intrinsic mechanisms that could result in long-lasting suppression (Benda and Herz, 2003; Gollisch and Herz, 2004; Ingham and McAlpine, 2004). In models of the IC, after hyperpolarization currents mediated by calcium-gated potassium channels have successfully predicted response to time-dynamic binaural stimuli. The rodent IC shows six distinct cell types, each exhibiting unique potassium currents, including delayed-rectifier and calcium-dependent K+ channels (Bond et al., 1999; Stocker and Pedarzani, 2000; Sivaramakrishnan and Oliver, 2001; Womack and Khodakhah, 2003). Additional mechanisms resulting in slow recovery of fast sodium channels have been suggested in models of adaptation, such as voltage dependent, high threshold potassium currents and voltage-dependent potassium channels (M-type) (Cai et al., 1998a,b; Benda and Herz, 2003).

Stimulus-specific adaptation (SSA), an adaptation to the stimulus history and not the history of activation (Ulanovsky et al., 2004; Briley and Krumbholz, 2013) has been observed in the ICx for sounds of different frequencies (Reches and Gutfreund, 2008); however, SSA was not observed in the ICx when spatial cues were tested (Gutfreund and Knudsen, 2006; Reches and Gutfreund, 2008; Netser et al., 2011). The mechanism of SSA has not been conclusively elucidated, although synaptic mechanisms have been implicated (Ulanovsky et al., 2004).

**Figure 3:**
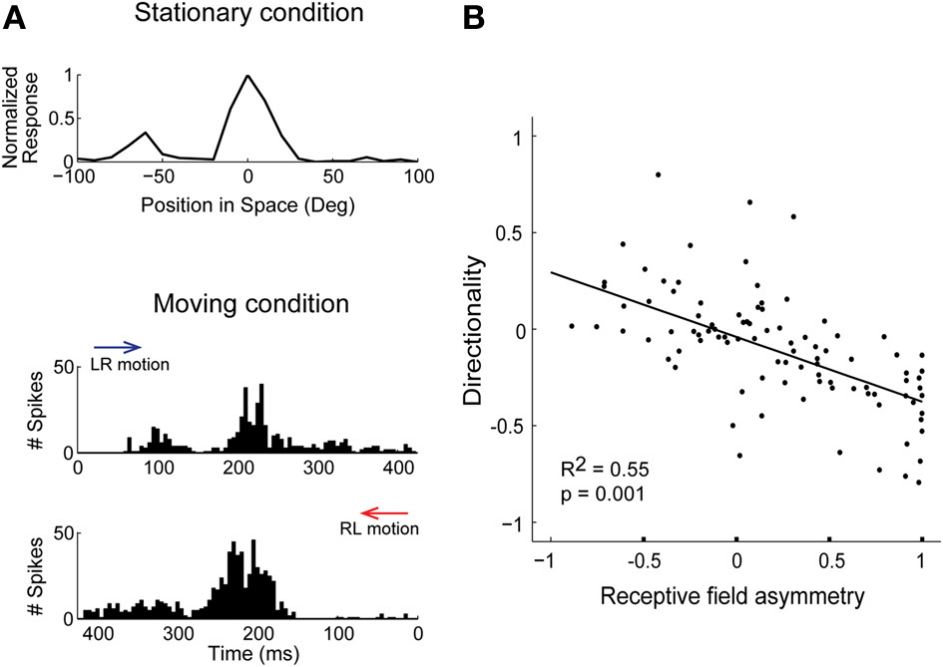
**Response history predicts direction selectivity in ICx. (A)** Spatial tuning in the horizontal plane measured with sound bursts presented in no particular order (stationary condition). The response is asymmetric around the center, displaying a larger response to the left. Bottom, during moving sounds, the response varied with direction. Motion in the left-to-right direction (LR, blue arrow) induced a less robust response at the center than the right-to-left direction (RL, red arrow). **(B)** Asymmetry of receptive fields was correlated with the preferred direction and strength of direction selectivity. Directionality and asymmetry are represented using normalized indices. Modified from Wang and Pena (2013).

If the response varies at different locations around the receptive-field center, i.e., spatial receptive fields are asymmetric, neurons can more strongly adapt stimuli in one direction than another, leading to selectivity for motion-direction. Unlike surround suppression, adaptation could induce direction selectivity in space-specific neurons that are not topographically arranged, as only receptive field asymmetry is required (Ingham et al., 2001). In support of this idea, ICx cells with asymmetrical spatial receptive fields were direction selective (Figure 3A) and their responses during sound motion could be predicted by the shape of the receptive field and their adaptation properties (Wang and Pena, 2013). At the population level, there was a direct relationship between receptive field asymmetry and the degree of direction selectivity (Figure 3B; Wang and Pena, 2013). The response to moving sounds at different velocities represents further evidence in support of adaptation. Directionality is stronger for fast moving sounds where short intervals between sounds at different locations induced robust adaptation (Wagner and Takahashi, 1992; Wang and Pena, 2013). Hence, neurons displaying response adaptation and asymmetric receptive fields automatically became direction selective without invoking network mechanisms for motion detection (Hassenstein and Reichardt, 1958; Barlow and Levick, 1965).

## Effect of map distortions on computations

Ramon y Cajal first proposed topographic organization was more metabolically efficient for wiring neural representations that mirrored the environment (Cajal, 1999). Maps are convenient for integrating over space and time since stimulus-driven activity varies systematically across the map in both dimensions. In the rat barrel cortex, local lateral connections are required for temporal coordination of whisking kinematics (Gao et al., 2003). In vision, computation of contrast and direction is also based on local connections (Barlow and Levick, 1965; Bloomfield and Xin, 2000; Völgyi et al., 2002; Zhou and Lee, 2008). Recent work showed that manipulating the spatial pattern of excitation within V1 can distort processing of visual shapes (Michel et al., 2013), indicating topography is exploited in processing high-level features.

In the auditory sensory modality, maps of space are found in close proximity to areas where motor output of orienting behaviors originate, such as in the brachium of the IC and the superior colliculus in mammals (Schnupp and King, 1997; Slee and Young, 2013) and in the ICx and OT in birds (Knudsen and Konishi, 1978a; Knudsen, 1982). A map of auditory space may be useful for multisensory integration, such as in the avian OT (Knudsen, 1982; Hyde and Knudsen, 2001) and mammalian SC (King et al., 1988; Doubell et al., 2000), where visual and auditory spaces are aligned. Coherence between auditory and visual maps may also be important during development, when visual experience can strongly modulate the topography of the auditory map in SC (King, 1999) and OT (Knudsen, 2002).

**Figure 4:**
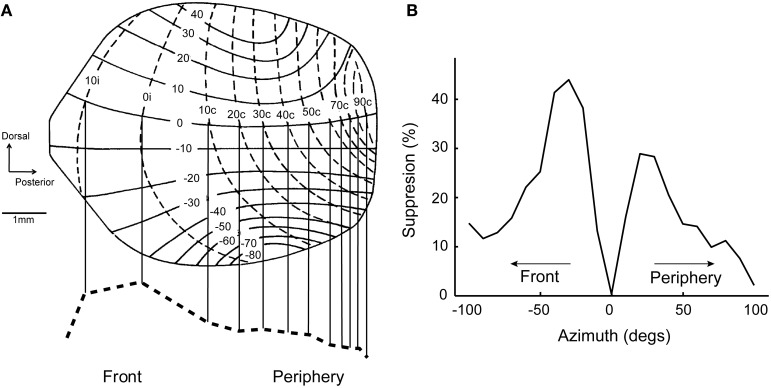
**Topography predicts population bias in surround suppression. (A)** Map of auditory space in OT modified from Knudsen (1982). Frontal space (±20°) is over-represented. Bottom, dashed line shows the estimated distribution of cells across the nucleus at zero elevation. **(B)** Surround suppression for a neuron tuned to 20° in the contralateral hemifield. The center of the receptive field is aligned to 0° on the x-axis. Suppression from frontal space (percent suppression from average response at the center) is stronger than from the periphery. Modified from Wang et al. (2012).

A common feature of neural maps is the distortion of the representation relative to the real world. Well-known examples include the over-represented visual fovea and the disproportionate homunculus in the somatosensory system, where ethologically important stimulus ranges are magnified in the brain (Penfield and Rasmussen, 1950; Azzopardi and Cowey, 1993). While these distortions may reflect the distribution of sensory afferents from the periphery, they often obey a hierarchy of sensitivity and discriminability requirements. In the ICx and OT maps of barn owls, locations in the front and below eye levels are over-represented (Figure 4A; Knudsen and Konishi, 1978a; Knudsen, 1982). This mapping may reflect orienting behavior, as owls face the target during pursuit (Payne, 1971; Hausmann et al., 2008) and descend from a height to capture prey (Volman, 1994). Distortions on the sensory surface are also consistent with the notion that space maps may represent a “place-coded probability distribution” (Knudsen et al., 1987; Fischer and Peña, 2011).

If there are more neurons representing the front, lateral connections coming from these cells could influence the response of neurons tuned to the periphery. Thus, the distortion in the ICx map could translate into a biased tuning at the population level. This was in fact the case; center-surround receptive fields showed a population bias where suppression from the front was stronger (Wang et al., 2012). The suppressive effect of peripherally-tuned cells was relatively weak on neurons tuned to the front. This bias in surround suppression resulted in a preference of ICx neurons for sounds approaching the front, since suppression is relatively weak in this direction (Figure 4B). Thus, receptive field shape and directional preference depended on tuning eccentricity in ICx, in a manner consistent with the distortions in the spatial map.

Space-specific neurons are commonly observed in the visual (Barlow and Levick, 1965; Hubel and Wiesel, 1974; Knudsen, 1982; Krapp and Hengstenberg, 1996), auditory (Knudsen and Konishi, 1978a; Brugge et al., 1994; Zhou and Wang, 2012) and somatosensory (Mountcastle, 1957; Simons, 1978) systems. Several of these regions are topographically-organized, non-uniform and exhibit center-surround receptive fields (Barlow et al., 1964; Livingstone, 1998; Drew and Feldman, 2007), as in the owl's ICx. Thus, biases in emergent responses that rely on center-surround integration may be a general property of sensory systems.

## Encoding sound motion

**Figure 5:**
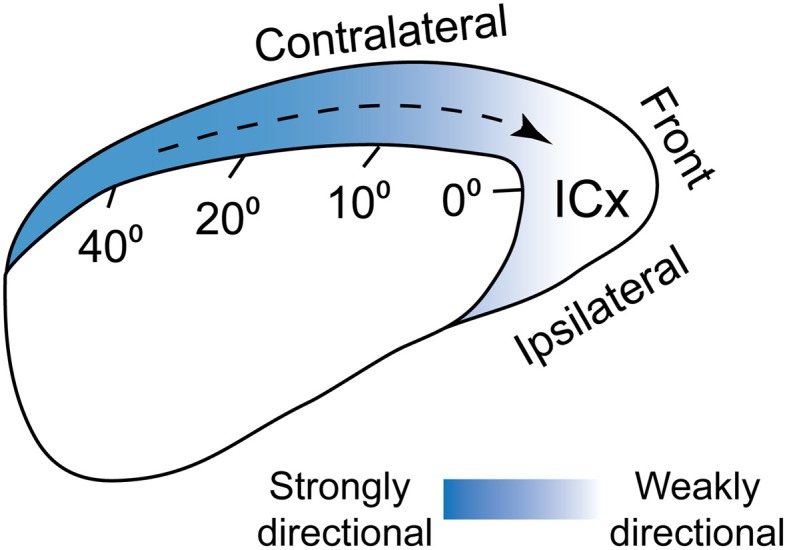
**Population-wide directional preference in ICx**. ICx neurons prefer sounds moving toward frontal space. This bias in directional preference is stronger for cells tuned to peripheral space and could be mediated both by biased surround suppression and adaptation. From Wang and Pena (2013).

Direction selective neurons have been reported in the mammalian (Sovijarvi and Hyvarinen, 1974; Rauschecker and Harris, 1989; Ahissar et al., 1992; Stumpf et al., 1992; Toronchuk et al., 1992; Wilson and O'Neill, 1998; Ingham et al., 2001) and avian auditory systems (Wagner and Takahashi, 1990, 1992; Wang et al., 2012). However, whether acoustic motion is encoded separately from other features has not been elucidated. We described above two mechanisms, surround suppression and adaptation, which explained a preference for sounds approaching the front. In both cases, the preference became stronger for cells tuned to peripheral space (Figure 5). These mechanisms take effect at different spatial scales relative to the neurons' receptive fields. The effect of surround suppression was strongest in the receptive field troughs flanking the center, where cells do not normally respond. On the other hand, the suppressive effects induced by response history were elicited by stimulation further away from the center (40–80°). Although the underlying mechanisms are different, they both induce preference for the same direction (Wang et al., 2012; Wang and Pena, 2013). Thus, these mechanisms could work synergistically to convey directional preference for sounds entering frontal space.

A topography of direction selectivity overlapping the map of auditory space indicates that neural activity in ICx carries information about both the location and the direction of the motion of sounds. This leads us to suggest that both location and motion direction appear represented in the owl's midbrain. However, whether direction and location are decoded independently remains to be demonstrated. Evidence from human EEG suggest that they are processed separately (Ducommun et al., 2002).

The emergence of motion-direction topography in ICx was supported by two principles. First, surround suppression was biased, such that neurons selective for frontal locations more strongly suppressed neurons at peripheral locations. This could be achieved by the non-uniform representation of space (Wang et al., 2012). Second, spatial receptive fields displayed systematic asymmetry in order for adaptation to induce topographically-organized direction selectivity. A likely mechanism for this asymmetry is that gain at frontal locations is higher due to the filtering properties of the head (Keller et al., 1998). Direction-dependent gain could induce a stronger response at the front, making receptive field asymmetry that is correlated with spatial tuning (Wang and Pena, 2013). Further, cortical auditory spatial receptive fields are often broad and complex (Brugge et al., 1996; Jenison et al., 2001; Mrsic-Flogel et al., 2005; Zhou and Wang, 2012), which could provide the asymmetry necessary to induce direction selectivity via adaptation.

## Conclusions

We showed that topography can result in computational biases within neural maps. Neurons at different locations in ICx responded depending on contextual excitation in space and their response history. These mechanisms elicited selectivity for higher-order stimulus properties such as time-dependent stimulus location. Because lateral interactions, adaptation and non-uniform maps are general properties of sensory maps, these processes are likely present in other sensory modalities.

Biased direction-selectivity may be important for detecting auditory looming objects (Maier and Ghazanfar, 2007) or estimating time to collision (Peron and Gabbiani, 2009). Our findings address the salience of sounds moving toward the front. From the viewpoint of coding strategy, the front is where spatial acuity (Knudsen et al., 1979) and signal intensity gain (Keller et al., 1998) are highest. Behaviorally, the owl places its target in the front during pursuit (Payne, 1971; Edut and Eilam, 2004; Hausmann et al., 2008; Ohayon et al., 2008). The preference for sounds moving inward could enhance the orienting response to stimuli entering the most sensitive region for sound localization. In general, directional biases in tuning to stimulus temporal dynamics may be adaptive for more efficient implementation of ethologically-relevant behaviors.

### Conflict of interest statement

The authors declare that the research was conducted in the absence of any commercial or financial relationships that could be construed as a potential conflict of interest.
